# Three pre-vaccine responses to Covid-like epidemics

**DOI:** 10.1371/journal.pone.0251349

**Published:** 2021-05-13

**Authors:** Lai-Sang Young, Zach Danial

**Affiliations:** 1 Courant Institute of Mathematical Sciences, New York University, New York, NY, United States of America; 2 Institute for Advanced Study, Princeton, NJ, United States of America; Universidade Federal Fluminense, BRAZIL

## Abstract

This paper contains a theoretical study of epidemic control. It is inspired by current events but not intended to be an accurate depiction of the SARS-CoV-2 pandemic. We consider the emergence of a highly transmissible pathogen, focusing on metropolitan areas. To ensure some degree of realism, we present a conceptual model of the outbreak and early attempts to stave off the onslaught, including the use of lockdowns. Model outputs show strong qualitative—in some respects even quantitative—resemblance to the events of Spring 2020 in many cities worldwide. We then use this model to project forward in time to examine different paths in epidemic control after the initial surge is tamed and before the arrival of vaccines. Three very different control strategies are analyzed, leading to vastly different outcomes in terms of economic recovery and total infected population (or progress toward herd immunity). Our model, which is a version of the SEIQR model, is a time-dependent dynamical system with feedback-control. One of the main conclusions of this analysis is that the course of the epidemic is not entirely dictated by the virus: how the population responds to it can play an equally important role in determining the eventual outcome.

## Introduction

The Covid-19 pandemic brought about renewed interest in epidemiological models. Medical research aside, vast numbers of modeling papers have been published since the start of the pandemic. We cite only a very small sample of these papers for illustration. The recent literature on Covid-modeling is vast, and as all this is happening in real time, it is not always easy to evaluate the impact of individual papers. Some of these papers have documented or analyzed events in specific regions of the world, others proposed mathematical models on how the infection spreads, yet others have tackled problems such as testing and herd immunity [1–10]. This paper contains a theoretical study of time evolutions of Covid-like epidemics from outbreak to vaccine arrival. We are interested in *controlling* the epidemics, i.e., in the population’s responses to the epidemic and the consequences of these responses. Our study goes beyond the initial outbreak to the aftermath of the onslaught, to examine the choices as society confronts the challenges of economic recovery and preventing a resurgence of the virus. We describe three different response strategies and track their performance over time.

One can divide epidemiological models into the following three broad categories (and combinations thereof): The first are *predictive models* (see e.g. [3, 11, 12]). Through data analysis and curve-fitting, these models tell us what will happen in the near-term based on knowledge of prior events. They are not aimed at theoretical understanding, but at providing guidance in real time in practical situations. The second kind are *agent-based models* (see e.g. [1, 13, 14]). These highly detailed mechanistic models offer vivid simulations of the dynamics of the infection process, but require large numbers of parameters on which model behavior depends, and are generally too complex for mathematical analysis. The third category consists of *conceptual* or *phenomenological models* (as in, e.g., [2, 15–17]). These idealized models seek to advance understanding through analysis, trading some amount of realism in exchange for conceptual insight. The models studied in this paper are of this third kind.

Our setup is entirely inspired by the SARS-CoV-2 pandemic of 2020, and our models have tried to capture some of its more salient features, from properties of the virus to social behaviors to shutdowns and to the resumption of activity, but we do not pretend that our models are an accurate depiction of what happened in any part of the world in 2020 nor were they intended as such. We have made a number of idealizations to achieve conceptual clarity and mathematical analyzability, while making sure not to deviate too much from reality. This is why we have described our models as responses to “Covid-like” epidemics. As we will show, the mathematical framework we offer is in fact flexible, hence exportable, and can be modified to describe a much larger class of epidemics than Covid-19.

Our mathematical models, which are infinite-dimensional time-dependent dynamical systems, have their origins in the usual SIR models [18–20], to which we have made very substantial modifications. The most significant difference is that we do not model the epidemic as an autonomous dynamical system. We focus on the population’s responses and consequences of the actions taken, modeling the time course of the epidemic as a dynamical system with *feedback-control*. Examples of control mechanisms include the following: We allow *r*, the mean number of individuals infected by each infectious host, to vary with time. More precisely, *r* is a function of density of contact and attack rate per contact, and these quantities are functions of time (e.g. facial coverings decrease attack rate, working from home decreases the amount of contact). The fraction of infectious individuals identified and removed from circulation is another control parameter. Central to our feedback-control mechanism is a quantity we call *δ*(*t*), representing the fraction of normal activity permitted to go on at time *t*; in lockdowns, this quantity is decreased to *δ*(*t*) = 0.3 (representing essential activity that must continue) and the pace at which it is brought back up to 1 represents the pace of reopening. In the model, all of these control quantities can be altered at will, and our aim is to understand the consequences of the choices made. The three post-lockdown, pre-vaccine scenarios discussed differ precisely in these control parameters.

Another important feature of the model is that quantities such as *E*(*t*) and *I*(*t*), fractions of the population that are exposed, respectively infectious, at time *t*, are treated as “hidden variables” to policy makers. For infectious diseases with a large amount of asymptomatic transmission such as Covid-19, the number of new daily cases that one reads about in the news can be deceiving. In recognition of that, we distinguish between groups that are “actually infectious” and those that are “known to be infectious”, and it is the size of the latter that serves as the basis for determination of control protocols.

To demonstrate resemblance to reality in our models and parameter choices, we present detailed model outputs during the first six or seven months following the outbreak. Our results show remarkable qualitative—even quantitative—resemblance to the data and trends in New York City. Using the same models, we then proceed to study the time course of the epidemic for another year or so, until vaccination changes the dynamics. Our results are not intended as predictions like those given by data-driven models; they are theoretical results aimed at clarifying the causes and effects of different strategies in epidemic control.

A number of papers with which our paper has overlap came to our attention after we had largely completed this work (all this is happening in real time!). One is [21], which reports on discussions of global exit strategies from a workshop held in 2020. The issues raised had much in common with those addressed in this paper, a difference being that their aim was to identify questions that needed to be addressed whereas we present concrete models for how some strategies might work. Another related paper is [7], the author of which focused on post-lockdown scenarios in France, and fitted parameters (similar to ours) with data, while ours is a general, theoretical study of control strategies inspired by those practiced in different parts of the world, covering the period that spans the first wave of infection to arrival of vaccine.

## Methods

We first introduce the underlying model used throughout the paper, leaving unspecified a number of model parameters and functions the selection of which will make the responses differ.

### An epidemic model with no control mechanisms (SEIR)

In this standard SEIR model (see [20]), we consider a random network of *N* nodes; each node represents a host, and nodes that are linked by edges are neighbors. The density of connections *m* at each node is defined to be $\frac{\langle k\rangle }{N}$ where 〈*k*〉 is the mean degree at a node. Each host is in one of four discrete states: healthy and susceptible (*S*), exposed (*E*), infectious (*I*), and recovered (*R*). Infectious hosts infect their susceptible neighbors at rate *β*, causing them to move from *S* to *E*. An exposed node becomes infectious $\tilde{\sigma }$ units of time (latency periods) later, and after becoming infectious, it recovers at rate *γ*. Upon recovery, a node acquires immunity which decays at rate $\tilde{\alpha }$. When immunity is lost, the node returns to state *S*.

We assume the total population is constant, and let *S*(*t*), *E*(*t*), *I*(*t*) and *R*(*t*) denote the fractions of susceptible, exposed, infectious and immune nodes at time *t*, so that *S*(*t*) + *E*(*t*) + *I*(*t*) + *R*(*t*) ≡ 1. Assuming that links between the infectious and susceptible nodes are uncorrelated, we obtain, by moment closure, the following system of delay differential equations (DDE) in the continuum limit as *N* → ∞:

$$\begin{array}{ccc} \dot{S}\left(t\right) & = & -\beta mS\left(t\right)I\left(t\right)+\tilde{\alpha }R\left(t\right),\\ \dot{E}\left(t\right) & = & \beta m[S\left(t\right)I\left(t\right)-S\left(t-\tilde{\sigma }\right)I\left(t-\tilde{\sigma }\right)],\\ \dot{I}\left(t\right) & = & \beta m\left[S\left(t-\tilde{\sigma }\right)I\left(t-\tilde{\sigma }\right)\right]-\gamma I\left(t\right),\\ \dot{R}\left(t\right) & = & -\tilde{\alpha }R\left(t\right)+\gamma I\left(t\right)\;. \end{array}$$

The equations above describe the transfer of mass among the four compartments of the model, and are known to generate an infinite dimensional dynamical system on a Banach space [22, 23]. As we are free to select a time unit, we will assume from here on that the recovery rate is 1. Latency periods and rate of immunity loss are then scaled accordingly, to $\sigma =\tilde{\sigma }\gamma$ and $\alpha =\frac{\tilde{\alpha }}{\gamma }$.

**Fig 1:**
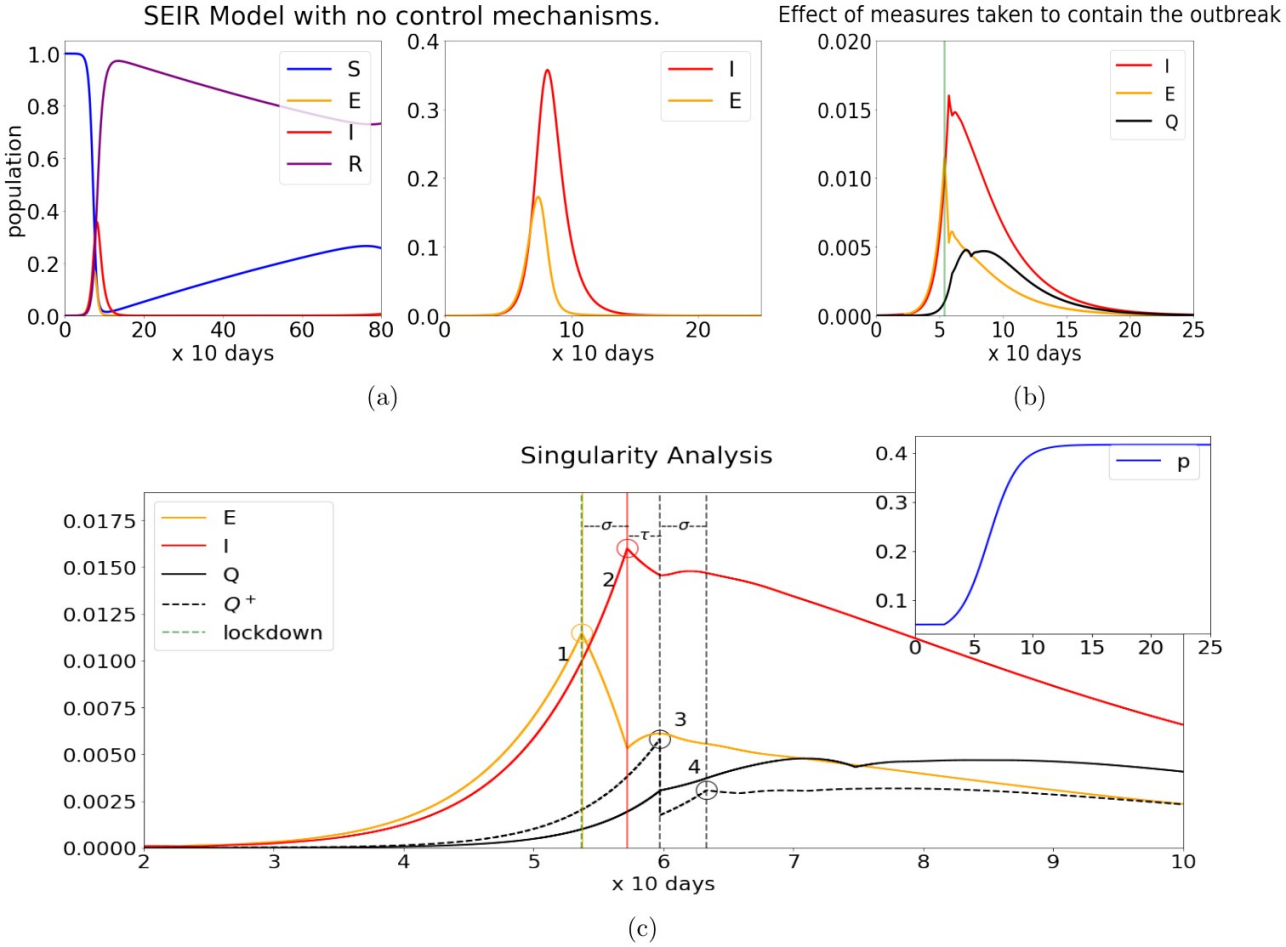
Basic model of early developments with and without control mechanisms. The left panel of (a) shows the development of an uncontrolled epidemic over the course of 800 days, simulated using the SEIR equations. The right panel of (a) takes a closer look at the curves for the [E]xposed and [I]nfected population. (b) shows the effect of a sustained lockdown and of isolating known infectious hosts [Q]; these efforts severely stunt the growth of the infection, lowering the peak of the infectious population from ∼36% to ∼1.6%. Plot (c) takes a detailed view of the singularities caused by the lockdown, which cascade through the system offset by the respective delay. The vertical green lines in (b) and (c) denote the time when alarms are raised (*t*_1_, dashed) and when the lockdown begins (*t*_2_, solid). Points of interest marked 1–5 in (c) are discussed in the text. The inset plot shows how testing ability (*p*) increases to its maximum over the course of 10 periods.

An example of the dynamics of this system of delay differential equations (DDE) with *r* = *βm* = 5, *σ* = 0.35 and *α* = 0.005 is shown in Fig 1(a). Here, the reproductive number *r* (which is very high) causes a strong wave of infection that eventually dies out after the pool of susceptible hosts drops below a certain critical value. This does not preclude the resurgence of the infection at a future time because immunity, though fairly long, is finite in time; a second wave will follow eventually though not during the time interval considered.

### Control parameters and functions

Following [17, 24], we add an isolation protocol as such: If a host remains infectious for *τ* units of time without having recovered, with probability *p* it enters a new state *Q* representing nodes that are isolated. The value *τ* represents the delay in getting tested and diagnosed. The hosts that do not enter state *Q* after *τ* units of time remain infectious until they recover on their own. A host in state *Q* does not infect others; it remains in this state for *κ* units of time, at the end of which it joins the recovered class *R*. Infectious hosts that are asymptomatic are not likely to be isolated, so the amount of asymptomatic transmission automatically puts an upper bound on *p*, the probability that an infectious individual will be isolated. Versions of an SEIQR model have been used in a number of epidemiological studies, e.g. [10, 25–28].

In addition to isolation, many other mechanisms were employed in the fight against Covid-19. Behavior such as frequent washing of hands and the wearing of masks have reduced the transmission rate *β* significantly since the early days of the infection. Social-distancing and the self-isolation of more susceptible groups, not to mention stay-at-home orders, can reduce *m*. (Similar ideas have been used in [29, 30]; how changes in *β* and *m* contribute to the time-dependence of *r* was also recognized in [21]). The ability of most localities to test and isolate infected hosts has greatly increased over time, thereby raising *p*. As *β*, *m* and *p* depend on human behavior and response capabilities, and these behaviors and capabilities evolve, we will model these three quantities not as fixed constants but as functions of time.

We further introduce the following two important quantities, both functions of time: The first is *Q*^+^(*t*), the fraction of the population that is diagnosed and isolated per unit time. It is the analog of the *daily new cases* reported in the news. We view *Q*^+^(*t*) as a known quantity on which responses will depend; quantities like *E*(*t*) and *I*(*t*), which describe the actual infection process, are largely hidden and unknowable explicitly in real time in epidemics such as Covid-19 that involve significant asymptomatic transmission. The second important quantity we introduce has to do with the loss of activity, economic and other kinds, due to the epidemic: we let *δ*(*t*) ∈ [0, 1] denote the fraction of normal activity that takes place at time *t*, so that *δ*(*t*) takes a plunge during the lockdown, and reopening is modeled by an increase in *δ*(*t*). We assume—as a simplification—that the amount of contact is proportional to *δ*, so that with reduced activity, the density of contact becomes *δm*.

Remembering that *β*, *m*, *p* and *δ* are functions of time, the following equations describe the basic model used in the rest of this paper:

$$\begin{array}{ccc} \dot{S}\left(t\right) & = & -(\delta \beta mSI)(t)+\alpha R(t) \end{array}$$ (1)

$$\begin{array}{ccc} \dot{E}\left(t\right) & = & \left(\delta \beta mSI)(t)-(\delta \beta mSI)(t-\sigma \right) \end{array}$$ (2)

$$\begin{array}{ccc} \dot{I}\left(t\right) & = & \left(\delta \beta mSI\right)\left(t-\sigma \right)-I\left(t\right)-Q^{+}\left(t\right) \end{array}$$ (3)

$$\begin{array}{ccc} \dot{Q}\left(t\right) & = & Q^{+}\left(t\right)-Q^{+}\left(t-\kappa \right) \end{array}$$ (4)

$$\begin{array}{ccc} \dot{R}\left(t\right) & = & I\left(t\right)+Q^{+}\left(t-\kappa \right)-\alpha R\left(t\right)\;, \end{array}$$ (5)

where *Q*^+^(*t*) is defined to be

$$\begin{array}{c} Q^{+}\left(t\right)≔e^{-\tau }p\left(t-\tau \right)\left(\delta \beta mSI\right)\left(t-\sigma -\tau \right)\;. \end{array}$$

The derivation of Eqs (1)–(5) is similar to those used in [17, 24], where *β*, *m* and *p* were assumed to be constant and *δ* ≡ 1. Specifically, (*δβmSI*)(*t* − *σ* − *τ*) is the rate at which mass is transferred from *E* to *I* *τ* units of time before *t*, and *e*^−*τ*^ *p*(*t* − *τ*) is the probability of identifying and isolating an infected node from the group transferred at time *t*.

This completes a description of the basic mathematical model used in the study of the three responses to a Covid-like epidemic which we describe next. For definiteness, we will fix the recovery rate *γ* to be 1/10, so that each unit of time in our simulations corresponds to 10 days. This means, in particular, that the daily new cases at time *t*, measured in terms of the fraction of the population diagnosed and isolated each day, is $\frac{1}{10}Q^{+}(t)$. The parameters *σ* and *τ* are also fixed at *σ* = 0.35 (or 3.5 days), *τ* = 0.25 (2.5 days), and *κ* = 1.5 (15 days), values that are roughly consistent with Covid-19. For example, for Covid-19 the onset of symptoms was found to be ∼5 days following exposure, while pre-symptomatic spread starts around 2 days before that [31, 32]. Our assumption of 3.5 days from exposure to becoming infectious is roughly consistent with these findings, as is our assumption that isolation—if the infected individual is isolated—tends to start on average around 6 days following exposure. Time-dependent parameters to be selected are *β*, *m*, *p* and *δ*.

Table 1 shows the list of parameters that appear in the model together with some values that are fixed throughout. We stress that this is a theoretical paper about generic epidemics caused by a highly transmissible pathogen. The parameters used, while inspired by Covid-19, are not intended for any one virus or situation.

**Table 1:** Table of parameters. Here as in equations and simulations in the rest of the paper, recovery rate is set to 1, so that each unit time interval [*t*, *t* + 1] corresponds to a 10-day period. The values below are normalized to this timescale.

Parameter name	Interpretation	Value
*β*	transmission rate	dynamic
*m*	contact density	dynamic
*p*	probability of isolation	dynamic
*δ*	activity level	in [0, 1], control variable
*σ*	incubation period	0.35 period
*τ*	delay in isolation	0.25 period
*κ*	isolation period	1.5 periods
*γ*	recovery rate	1 /period
*α*	rate of immunity loss	0.005 /period

## Results

All three of the models below describe the time course of a Covid-like epidemic in a fairly densely populated area, and the area’s efforts to keep the virus in check until the arrival of a vaccine. We have in mind a large metropolitan area with population upwards of a few million, as the assumption of homogeneity weakens when considering larger geographical regions. Our modeling of the infection-response dynamics in the first months after the outbreak is the same in all three models: it describes an all-out attempt to mitigate the onslaught, efforts crippled by unpreparedness and failure to comprehend the scale of the impending disaster. After fighting off the initial wave of infection, the challenges are, in some ways, more complex: resumption of activity and economic recovery entail a certain amount of risk of resurgence of the infection. The three models described herein differ in their responses to these conflicting goals.

### Outbreak and initial response

We begin with a description of how we have modeled the early months of the epidemic, the part common to all three models. Details below are not intended to be an accurate depiction of events in any one city or locale, but are inspired by what happened in New York City. A widely used website providing real-time statistics on the Coronavirus and other population data worldwide collected from government sources is Worldometer [33, 34]; another source containing detailed information about NYC and other US States and Territories is the CDC-maintained site [35]. For Covid-19, *r* (= *βm*) is often reported to be 2.0 − 2.5; see e.g. [36]. In hindsight it seems that the rate of asymptomatic transmission was not estimated correctly. The exact extent of asymptomatic transmission is still not known, though it is now generally accepted that up to half of the transmissions were likely undetected. Also, in a densely populated area, the number of contacts could be higher. We will assume a high initial spread rate of *r* = 5, stressing again that this rate changes with time, as it reflects not just properties of the virus but also human behavior.

At time *t* = 0 in our model, a small infection is quietly building but the threat is not yet recognized; think early February, 2020, in New York City. The early time course of the epidemic will be punctuated by three main transition points: *t*_1_, when the threat of an impending epidemic is first recognized; *t*_2_, when a lockdown is imposed to prevent the healthcare system from being overwhelmed; and *t*_3_, the start of the reopening.

For comparison with Fig 1(a), we show in Fig 1(b) the mitigating effect of the lockdown (without reopening, which differs from model to model), zooming in to see details more clearly in the first 100 days in Fig 1(c). These figures are results of simulations of Eqs (1)–(5), with the following parameters: we assume *r*(0) = *β*(0) ⋅ *m*(0) = 5, and *p*(0) = 0.05, the smallness of *p*(0) reflecting the difficulty in getting tested in those early days. These parameters are unchanged up to *t* = *t*_1_. At *t*_1_ = 2.5 (end of February in NYC), *r*(*t*) begins to fall as people start to take greater precautions in their personal behaviors. This decline continues for some time until it reaches a value of *r*(*t*) = 3.5 at *t* = 6.5, mirroring the fact that in response to Covid-19, precautions taken by individuals to reduce transmission rate (*β*) occurred gradually over time, e.g. face masks came later than handwashing. Likewise, direct contact (*m*) falls during the same period, as practices such as social-distancing and working from home are adopted. The parameter *p*(*t*) is also raised from 0.05 to 0.425, the steep part of the rise representing the scramble to improve testing capability and to secure the infrastructure and personnel needed to isolate and care for infected hosts.

An important feature of our model is that the measures taken by health authorities in these first months are based solely on *Q*^+^(*t*), the quantity analogous to “daily new cases”. Up until around *t* ∼ 3, *Q*^+^(*t*) is barely visible, but it rises rapidly thereafter, raising concern that demand for hospital beds may soon exceed healthcare capabilities. These concerns eventually leads to a lockdown, which occurs in our simulations at *t*_2_ ≈ 5.4, when *Q*^+^(*t*) reaches 0.002, or 2000 new cases/day in a city of 10 million; in NYC, this happened on March 23, 20–30 days after the initial alarm. In the model, the lockdown is described by lowering *δ*(*t*) from 1 to 0.3, a fraction representing the essential activity (such as food production and health-related maintenance) that must continue.

Observe in Fig 1(c) the effects of the lockdown at time *t*_2_ as they cascade through the system: First there is a sharp change in derivative in the Exposed (E) category immediately following the lockdown (the point marked “1”). Even as the fraction in E is decreased, *Q*^+^(*t*) continues to rise very substantially: *I*(*t*) does not turn around until *σ* units of time later, *σ* being the incubation period (the point marked “2”); a drop in *Q*^+^(*t*) is not seen for another *τ* units of time, *τ* being the average duration for an infected individual to be isolated if they are isolated (the point marked “3”). This sudden drop in *Q*^+^(*t*) is a reaction to the lockdown; community spread does not abate until after *I*(*t*) turns around, and this is reflected in the change in slope of *Q*^+^(*t*) another *σ*+*τ* units of time later (the point marked “4”). Notice that after the significant drop, *Q*^+^(*t*) continues to rise between the points marked “3” and “4” even as *I*(*t*) is decreasing. This occurs because *p*(*t*) is increasing: when testing is more readily available, a larger fraction of the infected becomes known, masking some of the progress made in reducing the rise of infection.

A striking feature of these simulations, based entirely on Eqs (1)–(5), is the dramatic difference between the actual numbers of people infected and the numbers confirmed positive in these early months of the epidemic. This happens because *r* is large and *p* is small, especially early on, allowing infected individuals to circulate in the general population undetected. Indeed as we will see, even after these early months, *Q*^+^(*t*) is still not indicative of the actual number of infected due to nontrivial asymptomatic transmission. The eventual value of *p*, which is quite far from 1, reflects the presence of undetected asymptomatic transmission (as well as the failure of a small fraction of those known to be infected to isolate themselves).

### Model 1: Steering a steady course between controlled infection and resumption of activity

We assume that initial developments including the lockdown are as above. Model 1 describes the theoretical possibility of judiciously adjusting the amount of reopening to maintain a steady infection rate. More precisely, sometime after the lockdown, *Q*^+^(*t*) will start to decrease. We fix a value $Q^{+}\left(t\right)=Q_{\text{open}}^{+}$, at which point reopening begins. In this model, the value $Q_{\text{open}}^{+}$ is assumed to be an acceptable rate of infection as trade-off for economic recovery. We propose an algorithm to steer a course that permits the maximum amount of activity without allowing *Q*^+^(*t*) to exceed $Q_{\text{open}}^{+}$. Such a course will be maintained until a vaccine becomes available, with as little disruption as possible in the form of opening and reclosing.

The algorithm for steering a steady course after reopening is motivated from [17, 24], where it was shown in that simpler context that the isolation scheme lowers *r* by a factor of (1 − *pe*^−*τ*^), i.e., whether an incipient outbreak will develop into a full-blown epidemic depends on whether *βm*(1 − *pe*^−*τ*^) is less than 1. This is under the assumption that the entire population participates in the disease dynamic. It was also shown that if a fraction *x* does not participate in the spreading of the disease, then the outbreak is contained if and only if

$$\begin{array}{c} \beta m\left(1-x\right)\left(1-pe^{-\tau }\right)<1\;. \end{array}$$ (6)

To steer a steady course after reopening, we propose the following algorithm for *δ*(*t*), the fraction of normal activity permitted: if $Q^{+}\left(t\right)\geq Q_{\text{open}}^{+}$, we set *δ*(*t*) = *δ*(*t* − *Δt*) where *Δt* = 0.005, i.e., reopening is paused if *Q*^+^(*t*) is not below the desired value; and if $Q^{+}\left(t\right)<Q_{\text{open}}^{+}$, then we let *δ*(*t*) be determined by

$$\begin{array}{c} \delta \left(t\right)\cdot r_{\text{eff}}(t-\frac{1}{2})=1 \end{array}$$ (7)

where

$$\begin{array}{c} r_{\text{eff}}\left(t\right)=\left(\beta m\right)\left(t\right)\cdot \left(1-p\left(t\right)e^{-\tau }\right)\cdot \left(1-R\left(t\right)-Q\left(t\right)\right)\;. \end{array}$$ (8)

The definition of *r*_eff_(*t*) comes from (6); the fraction of population participating in the infection process in the short run is (1 − *R*(*t*) − *Q*(*t*)) as the loss of immunity is slow. Assuming further that the amount of contact is proportional to *δ*(*t*), we arrive at the equation *δ*(*t*)*r*_eff_(*t*) = 1 as the theoretical tipping point. In Eq (7), *r*_eff_ is evaluated at $t-\frac{1}{2}$, i.e. we have used its value from 5 days earlier, to simulate the planning involved in reopening.

**Fig 2:**
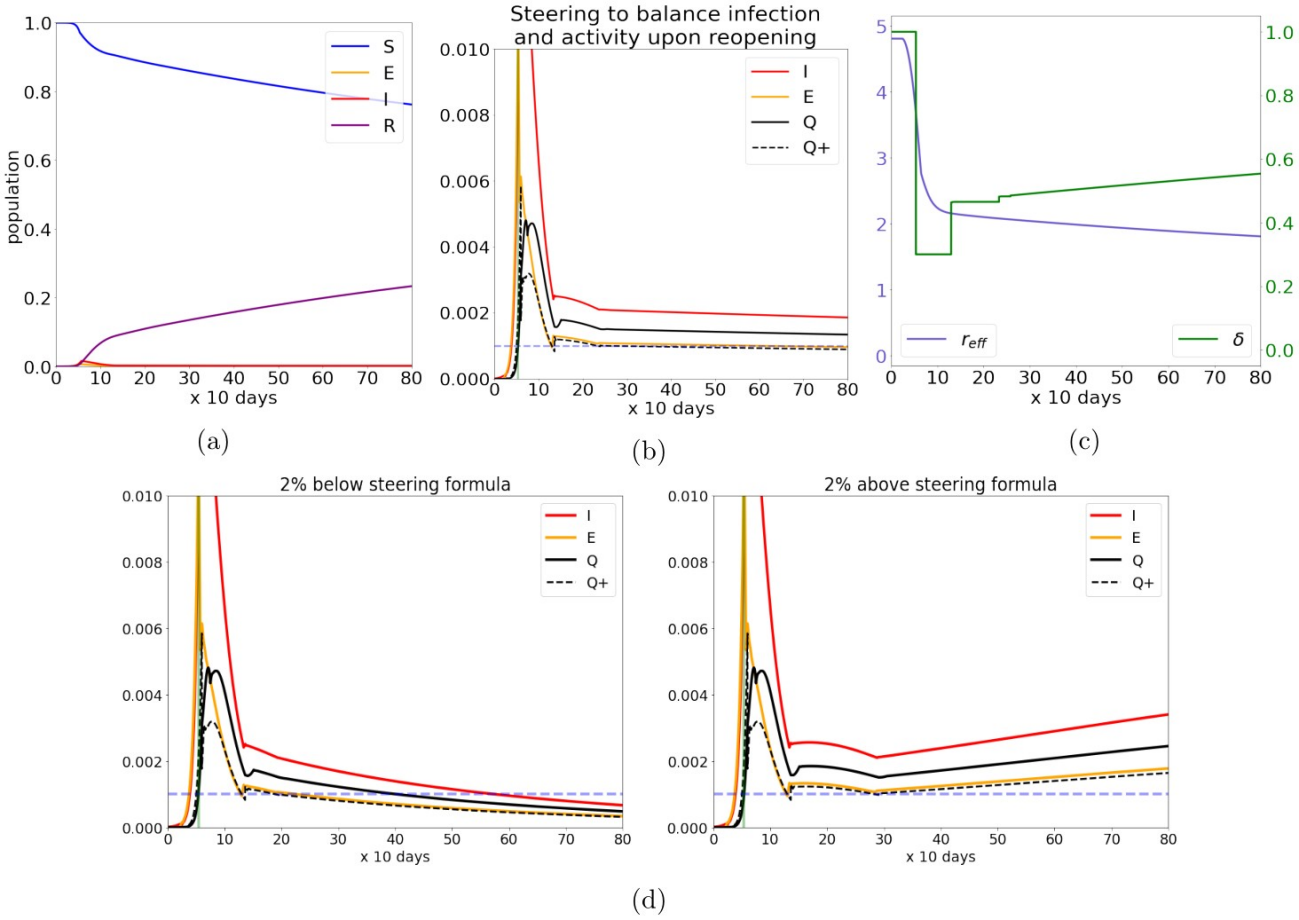
Steering to balance infection and activity post-lockdown. (a) and (b) show the development of the controlled epidemic as outlined in Model 1, with (a) showing a roughly linear rate of transfer from [S]usceptible to [R]ecovered and immune and (b) zooming in on the curves for the [E]xposed, [I]nfected and isolated population. Plot (c) shows the time-course of the control variable (*δ*, in green) and effective reproductive number (*r*_eff_, in blue). We can understand the epidemic response by following *δ*(*t*) in plot (c). After stunting the initial outbreak, the system is steered to maintain a constant level of infection. The plots in (d) showcase the sensitivity of the approximations used in the steering formula: steering at 2% above/below the prescribed formula results in growth/decay of the fraction exposed.

Fig 2a–2c show simulation results for $Q_{\text{open}}^{+}=0.001$. In a city of 10 million people, a fraction of 0.001 testing positive every 10 days corresponds to 1000 new cases/day. With this choice of $Q_{\text{open}}^{+}$, reopening in the model begins at *t*_3_ ≈ 13, a little over 75 days after the lockdown, a close comparison to the 80-day lockdown for NYC.

We remark that the validity of the formula in (7) should not be taken for granted: The classical SIR result [20], or its extension in [17, 24], asserts only that if parameters are constant, then *r* < 1 implies *I*(*t*) will tend to zero as *t* → ∞, that is, the system will in time tend to a disease-free equilibrium. The present situation differs from these earlier results in two important ways: First, here the system is not tending to a disease-free equilibrium; instead we are trying to steer it to a *nonequilibrium steady state*, where mass is continuously transferred among the different compartments in the system but the rates of transfer are constant. Second, we are not near this steady state at the beginning, nor do we give ourselves infinitely long to get there; we change *δ*(*t*) constantly to maximize the resumption of activity. What ensures the success of this algorithm is that we pause *δ*(*t*) whenever *Q*^+^(*t*) exceeds our target value, and with *δ*(*t*) paused, the rescaled effective reproductive number will drop below 1 sooner or later for as long as mass is transferred into *R*(*t*) faster than it is leaving. Pausing *δ*(*t*) happens only initially; once the system is sufficiently close to a steady state, the near-constant rate of transfer from *S*(*t*) to *R*(*t*) will ensure that *δ*(*t*) rises steadily.

Fig 2(d) confirms the efficacy of this algorithm: we show that if we steer at 98% of the estimated *δ*(*t*), *I*(*t*) will tend to zero, whereas if we steer at 102% of the estimated value, infection will grow.

We have used $Q_{\text{open}}^{+}=0.001$ or 1000 new cases/day in a city of 10 million people for illustration, but the same strategy (with minor adjustment) works in principle for any value of $Q_{\text{open}}^{+}$. For the State of New York (which has a population of just under 20 million), the first several months since reopening saw a steady rate on the order of 800 new cases/day, with a plot of *Q*^+^(*t*) qualitatively remarkably similar to that in Fig 2(b) [34, 35]. It was hard to know how much of that was by design, though some level of steering was likely involved.

Returning to our model, the situation with regard to recovery at the end of two years (i.e. at *t* ∼ 70) for a steady state of $Q_{\text{open}}^{+}∼0.001$ is summarized in Fig 2(a) and 2(c). Though not without a human toll, this relatively low rate of infection does not stress the healthcare system, but return to normalcy is also slow; at the end of two years the feasible amount of activity is still not much more than 50% (Fig 2(c)), and progress toward herd immunity is also very modest (Fig 2(a)).

We discuss next a different strategy, one that places greater emphasis on economic recovery.

### Model 2: Living with the virus

Unlike Model 1 with a low value of $Q_{\text{open}}^{+}$, Model 2 places greater emphasis on the resumption of activity accepting the risks of higher infection rates.

The viability of this strategy is predicated on strong testing capabilities and medical advances, on the availability of therapeutics to minimize serious illnesses and death. For Covid-19, these advances have been remarkable: a great deal has been learned during the first six months after the initial outbreak on how to mitigate the effects of the infection, and how to tamp down overreactions of the immune system. As a result, death rates among hospitalized Covid patients have been reduced significantly. At the same time, economic losses during the lockdown and subsequent partial reopening have been staggering, and many are experiencing quarantine fatigue half a year into the pandemic. Increasingly in the western world, there is growing resistance from the general population to further restrictions on their social behaviors, raising the issue of whether keeping infection low at all costs is the best option forward. An alternative is to accept some degree of risk in pursuit of a faster return to normalcy.

For Covid-19, the risk of adverse effects (such as inflammation of the heart and chronic fatigue) post-infection have been documented, and the soundness of this “living with the virus” strategy from the medical standpoint remains unclear. But our goal is not to evaluate the soundness of this strategy with respect to this or any specific virus. We are interested in the impact of such a strategy on activity retained and progress toward herd immunity.

Model 2 is identical to Model 1 through the lockdown, which we assume is partially lifted when *Q*^+^(*t*) drops down to $Q_{\text{open}}^{+}=0.001$. It is from this point on that the models diverge. We describe below two examples of how one might live with the virus without relinquishing control:

#### Strategy 2A: Resetting *Q*^+^(*t*) after a recovery period

This strategy calls for maintaining *Q*^+^(*t*) at 0.001 upon reopening for a period of 2–3 months to allow time to recover from the initial onslaught, to improve testing capability and hospital preparedness, and build up the necessary stockpile of PPE (personal protective equipment). Once that is accomplished, *Q*^+^(*t*) would be allowed to climb, until a new, more desirable value of $Q_{*}^{+}$ is reached, and to maintain *Q*^+^(*t*) at this higher level as was done in Model 1.

**Fig 3:**
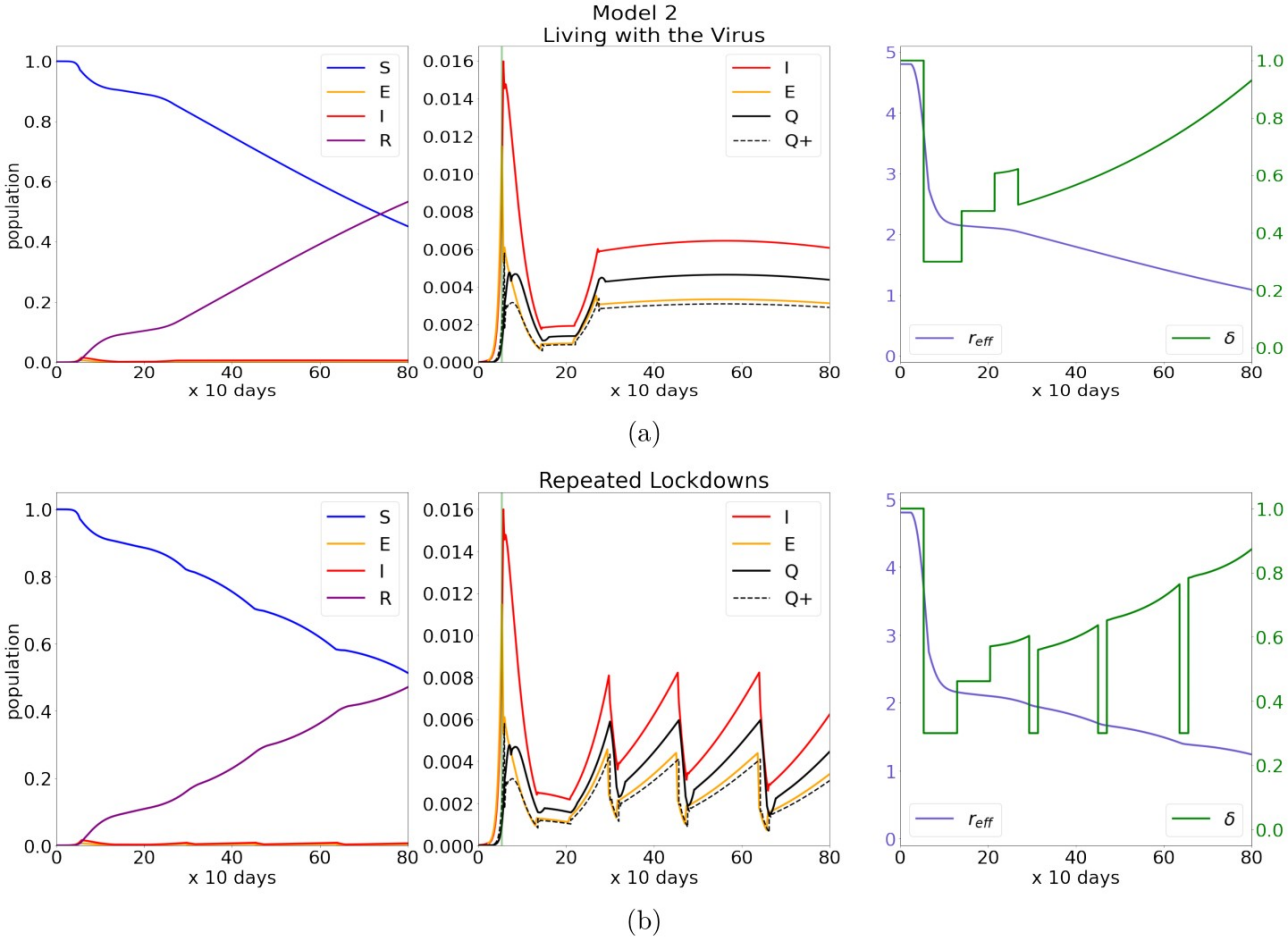
Two more aggressive reopening strategies. (a) and (b) show the time-course of the two controlled epidemic strategies outlined in Model 2. Plot (a) uses a controlled increase in the level of infection followed by maintaining steering at an elevated level, plot (b) features slowed outbreaks with periodic lockdowns. Notice the considerable build-up of immunity (*R* in (a), (b)) over the time period considered in the simulation. Also note that steering can be turned on and off to strategically adjust the level of infection. Maintaining the virus at such a high level, if possible, has a significant impact on both immunity build-up and the return to normal. In these cases *δ*(*t* > 25) has a positive second derivative, compared to Model 1 where *δ*(*t* > 25) seems to increase at a slow and almost linear rate.

Fig 3(a) illustrates this strategy. Upon reopening, the steering formula (7) was used to maintain *Q*^+^(*t*) at ∼0.001 for 7–8 units of time. We then relaxed *δ*(*t*), using a prescription such as

$$\begin{array}{c} \delta \left(t\right)\cdot r_{\text{eff}}(t-\frac{1}{2})=1.25 \end{array}$$ (9)

that will cause the infection rate to grow. After a period of controlled increase, in this case when *Q*^+^(*t*) reached 0.003, the rate of new infections considered to be a reasonable compromise, we switched back to Eq (7), maintaining this protocol from here on. That is to say, when *Q*^+^(*t*) reached 0.003, we reset *δ*(*t*) so that *δ* · *r*_eff_ = 1, in the hope of maintaining *Q*^+^(*t*) at around 0.003 from that point on using the steering formula (7). The left panel of Fig 3(a) shows a considerably larger fraction of the population with immunity compared to Model 1, and the right panel shows the stronger progress towards resumption of activity even after *δ*(*t*) has been dialed back. However, these gains are not without cost, as a higher *R*(*t*) also means a larger fraction of the population has been infected.

This example demonstrates the flexibility of the steering strategy—one can bring the level of infection up and down at will by adjusting the level of activity permitted. We cannot emphasize more that the course of the epidemic is not described by an autonomous dynamical system but one with feedback-control.

#### Strategy 2B: Periodic lockdowns

In this strategy, after a recovery period following the initial onslaught, we purposely set *δ*(*t*) so that *r*_eff_ · *δ*(*t*) is larger than 1. The rate of infection is allowed to grow, but slowly and in a controlled way. When it reaches a threshold considered “too high”, a brief lockdown of finite (predetermined) duration is implemented, with similar cycles of events to be repeated.

Fig 3(b) shows an example of such a strategy. Here *r*_eff_ · *δ*(*t*) is set to 1.2 at the end of a recovery period identical to that in Strategy 2A, and lockdowns of a maximum of 20 days are implemented when *Q*^+^(*t*) reaches 0.004, or 4,000 new cases/day in a city of 10 million people. In this example, the second lockdown occurs 7–8 months (200–240 days) after the first reopening. (This is comparable to events in Europe where the first lockdown was around March 10 and the second around Nov 1.) In subsequent cycles, no recovery period is needed, and we set *r*_eff_ · *δ*(*t*) = 1.1 to avoid too frequent lockdowns.

An important observation is that for as long as subsequent waves are controlled and not allowed to grow without bound, the lockdowns needed to control *Q*^+^(*t*) can be relatively short: in our example, a duration of 20 days at *δ* = 0.3 is adequate for reducing *Q*^+^(*t*) down to essentially the same level as the initial lockdown. As can be seen in the right panel, the initial lockdown lasted much longer, due to the large pool of infected hosts that accumulated during the early days of the epidemic. Strategies 2A and 2B have strikingly similar overall effects in resumption of activity and fraction of the population infected.

Some form of periodic lockdown—whether it was event-driven or by design—describes the evolution of *Q*^+^ in a number of west European countries (e.g. France, Italy) [33]: the initial lockdown in March 2020 was followed by a second one in November 2020 and a third one in March 2021, though the later lockdowns were not as severe than those depicted in Fig 3(b).

### Model 3: Zero-tolerance

Model 2 describes a response that permits a more aggressive path towards resumption of activity while accepting a larger rate of infection with its inherent risks. Model 3 describes yet another approach, namely that of zero tolerance. Zero-tolerance strategies seek an even faster return to normal activity *and* very low rates of infection at the same time. This strategy requires a certain amount of sacrifice, and perpetual vigilance. It is practiced in some Asian countries including China, as well as in e.g. Australia and New Zealand (see [33]). It is also practiced on a smaller scale within institutions and organizations, though we will focus on large municipalities. Below we describe how such a strategy could work applying the mathematical framework of this paper.

We assume in Model 3 that the initial stages of the epidemic are as in Models 1 and 2, except that reopening takes place only when *Q*^+^(*t*) reaches a very low value, a value one then seeks to maintain. Examples of $Q_{\text{open}}^{+}$ we have in mind are on the order of 10^−5^ to 5 × 10^−5^. In a city of 10 million people, 10^−5^ new cases/10 days translates into 10 new cases/day, a very ambitious goal. What makes such a goal viable alongside a bold step towards resumption of normal activity is that when the number of new cases is very small, there is an effective tool: *contact tracing*, something we have neglected in Models 1 and 2 because with hundreds to thousands of new cases a day, the effect of contact tracing is minimal. The smaller the caseload, the more effective this tool is to prevent epidemic spread.

We do not model contact tracing in detail, but use the following stand-in that we believe has a similar effect: Let *ϵ*(*t*) ∈ [0, 1] denote the fraction of infection averted at time *t*, i.e., we assume that among those who would have become infectious at time *t* in the absence of contact-tracing, a fraction *ϵ*(*t*) is quarantined before they infect anyone (whether or not these individuals get infected is immaterial, as long as they infect no one). The procedure we implement is as follows: At time *t*, instead of transferring the mass that entered category *E* at time *t* − *σ* from *E* to *I*, a fraction equal to *ϵ*(*t*) of this mass is transferred directly to *Q*, bypassing *I*. The remaining (1 − *ϵ*)-fraction of the mass is moved to *I* and dealt with as before. That is, Eqs (1)–(5) are modified to read

$$\begin{array}{ccc} \dot{S}\left(t\right) & = & -(\delta \beta mSI)(t)+\alpha R(t) \end{array}$$ (10)

$$\begin{array}{ccc} \dot{E}\left(t\right) & = & \left(\delta \beta mSI)(t)-(\delta \beta mSI)(t-\sigma \right) \end{array}$$ (11)

$$\begin{array}{ccc} \dot{I}\left(t\right) & = & \left(1-\epsilon \left(t\right)\right)\left(\delta \beta mSI\right)\left(t-\sigma \right)-I\left(t\right)-Q^{+}\left(t\right) \end{array}$$ (12)

$$\begin{array}{ccc} \dot{Q}\left(t\right) & = & Q^{+}\left(t\right)-Q^{+}\left(t-\kappa \right)+Q_{\epsilon }^{+}\left(t\right)-Q_{\epsilon }^{+}\left(t-\kappa \right) \end{array}$$ (13)

$$\begin{array}{ccc} \dot{R}\left(t\right) & = & I\left(t\right)+Q^{+}\left(t-\kappa \right)+Q_{\epsilon }^{+}\left(t-\kappa \right)-\alpha R\left(t\right)\;, \end{array}$$ (14)

where mass that exits *E* are now separated into two streams one of which, $Q_{\in }^{+}$, goes directly to *Q*:

$$\begin{array}{ccc} Q^{+}\left(t\right) & ≔ & \left(1-\epsilon \left(t\right)\right)e^{-\tau }p\left(t-\tau \right)\left(\delta \beta mSI\right)\left(t-\sigma -\tau \right)\;, \end{array}$$ (15)

$$\begin{array}{ccc} Q_{\epsilon }^{+}\left(t\right) & ≔ & \epsilon (t)(\delta \beta mSI)(t-\sigma ) \end{array}$$ (16)

This procedure is simple and captures the idea of contact tracing but causes *E*(*t*) to be higher than should be, in a way that is not consequential.

We assume that *ϵ*(*t*) depends on the number of daily cases. Consider, for illustration, a population of 10 million, and let

$$\begin{array}{c} \epsilon \left(t\right)=\frac{1}{2}\cdot {\left[1+\text{exp}(\frac{10^{6}\;Q^{+}\left(t-\frac{1}{2}\right)-100}{20})\right]}^{-1}\;. \end{array}$$

Here 10^6^ *Q*^+^(*t*) is the number of daily new cases, and the formula above implies that as *Q*^+^(*t*) → 0, $\in \left(t\right)\approx \frac{1}{2}$, a value considerably below 100% because of asymptomatic transmissions that are not easily detected, let alone traced. We assume also that *ϵ*(*t*) ≪ 1 when the daily new cases reach 200; this number could be larger or smaller depending on the workforce dedicated to the task and the degree of cooperation they receive. In any case the exact form of *ϵ*(*t*) is unimportant; the only features that matter are that success rate decreases monotonically to 0 as the number of cases increases, and it is strictly <1 even as *Q*^+^(*t*) → 0.

With this protocol in place, the quantity *r*_eff_ is further reduced from the formula in (8) to

$$\begin{array}{c} r_{\text{eff}}\left(t\right)=\left(\beta m\right)\left(t\right)\cdot \left(1-p\left(t\right)e^{-\tau }\right)\cdot \left(1-R\left(t\right)-Q\left(t\right)\right)\cdot \left(1-\epsilon \left(t\right)\right)\;. \end{array}$$ (17)

**Fig 4:**
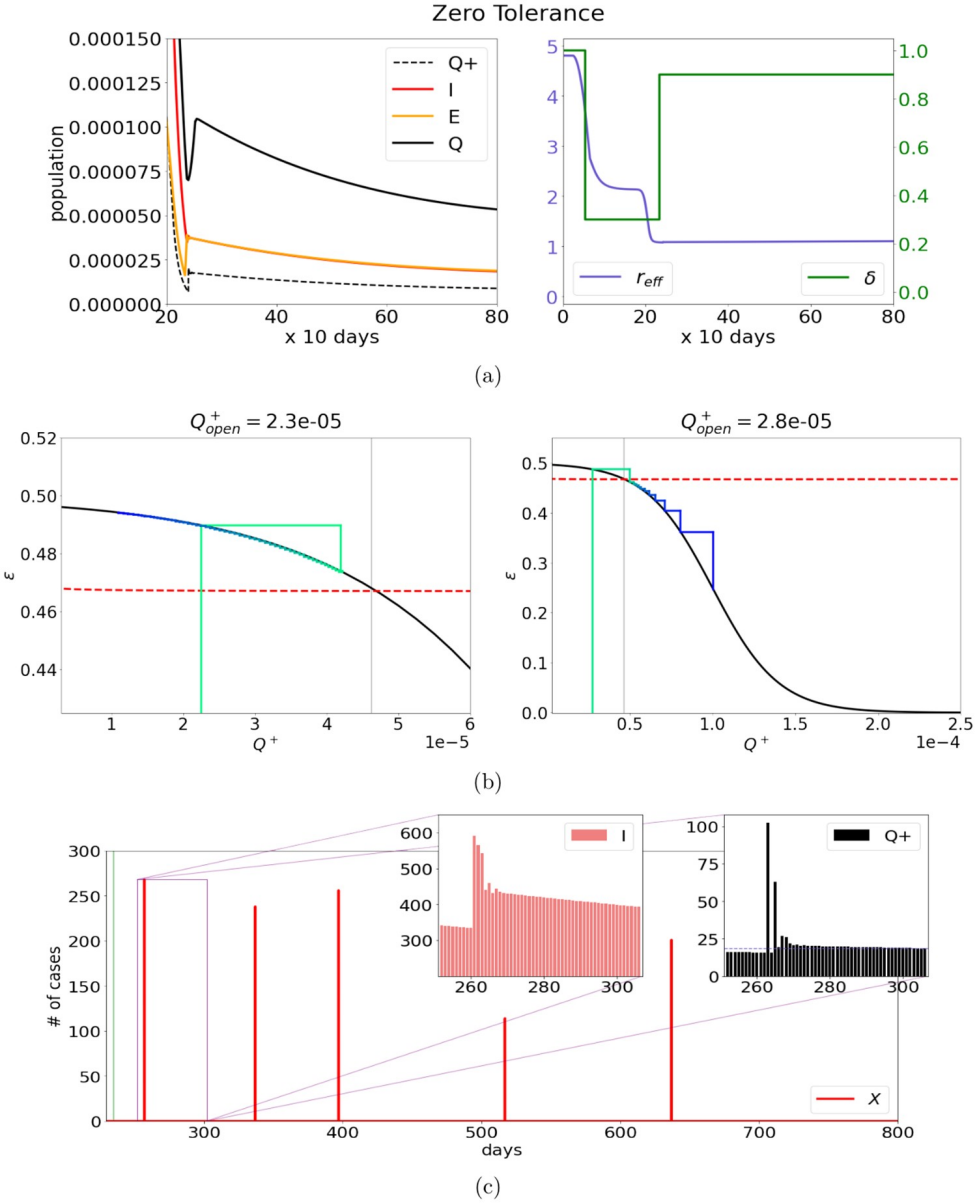
Zero tolerance: Instability and susceptibility to unforseen events. (a) takes a close look at the time-course of the [E]xposed and [I]nfected populations after reopening according to the Zero Tolerance strategy outlined in Model 3. After an extended lockdown brings the infection level down by several orders of magnitude, activity returns to 90% of the normal level without a drastic resurgence of infection. Plot (b) illustrates the instability of contact tracing. It shows the system’s trajectory through (*Q*^+^, *ϵ*) space, updated every period, with time increasing as the line color changes from green to blue. The black curve is the success of contact tracing (*ϵ*) for a given value of *Q*^+^ and the red dashed line is the *ϵ* required to inhibit an outbreak (numerically computed). To take advantage of contact tracing, reopening values of *Q*^+^ must be to the left of the grey vertical line. That, however, may not be sufficient: an outbreak can still occur due to inevitable rise of *Q*^+^ at the onset of reopening as shown in the panel on the right. Plot (c) features histograms showcasing the effect of stochastic spikes modeling events of unknown origin on *I* and *Q*^+^ by the number of cases. The green vertical line marks the reopening.

Fig 4(a) shows results of a simulation using the contact tracing protocol above. For illustration, we set *δ*(*t*)≡0.9. In this example, contact tracing is successful, and one is able to go back to 90% of normal activity immediately, a figure considerably higher than what is achieved in Models 1 and 2, and maintain a very small *Q*^+^(*t*) at the same time.

We finish with a discussion of two features of zero-tolerance strategies.

Our first observation is that relying on contact tracing to reopen to high levels of activity with only a small fraction of the population enjoying immunity is inherently unstable. For values of *δ* and *Q*^+^ for which *r*_eff_ · *δ* < 1, it is likely that *Q*^+^ will decrease. Since a smaller *Q*^+^ brings a larger *ϵ*, the situation improves, increasing the probability that the infection will further abate (Fig 4(b), left). Consider next increasing *Q*^+^ with *δ* fixed. Because *ϵ* goes down when *Q*^+^ goes up, and *r*_eff_ is proportional to (1 − *ϵ*), there is a threshold beyond which *r*_eff_ · *δ* will exceed 1. Once this threshold is crossed, more new infection will follow, causing *Q*^+^ to get larger still, and for a larger *Q*^+^, contact tracing becomes even less effective. The situation spirals out of control quickly, as depicted in Fig 4(b), right.

The discussion above tells us that given *Q*^+^(*t*), hence *ϵ*(*t*), one should choose *δ*(*t*) so that *r*_eff_ · *δ* < 1. But this may not be good enough: With *ϵ* indexed to *Q*^+^, a transient rise in *Q*^+^ can cause *ϵ* to dip and *r*_eff_ to rise, and once the threshold *δ* · *r*_eff_ = 1 is crossed, there is the potential for an outbreak; this is what happened in Fig 4(b), right. Due to the volatility of the situation, one must set *δ*(*t*) not just to have *r*_eff_ · *δ* < 1 but with enough leeway to prevent accidental crossing of the threshold.

This leads to our second observation, which has to do with accidents, or clusters of unknown origin, modeled here as random events. In our original network model, transmission occurs by chance. While mean field models are reasonable when *I*(*t*) is not so small, when the numbers are small stochastic effects can be significant. Large deviations can potentially lead to instabilities of the type discussed above. The question is: Does this happen easily, and is it a cause for concern?

We simulated in Fig 4(c) spikes of random sizes that occur as a Poisson time process, at a rate of one significant event every 100 days. In each event, the number of individuals infected is a random variable *X* taking values in [1, 300], uniformly distributed. Since these spikes are assumed to be surprises, they are not contact traced: all *X* individuals become infectious. As usual, *τ* units of time after they become infectious, a fraction *pe*^−*τ*^ is diagnosed and isolated—it is only at this time that the spike is discovered. Once discovered, we assumed, for definiteness, that half of the remaining infectious individuals are “traced” and isolated in another *τ* units of time.

It would seem that a spike of e.g. 250 new cases would trigger a major infection event, but surprisingly, it did not, and the insets in Fig 4(c) explain why: When ∼20 new cases are discovered each day, the number of infectious individuals circulating in the population is in fact >300, and the addition of 250 new cases does not raise *I*(*t*) or *Q*^+^(*t*) significantly above baseline values *provided the spike is resolved quickly.* That is, provided that the system has the ability to resolve spikes quickly, Model 3 appears to be quite stable with respect to stochastic events of this type.

We have not discussed contact-tracing under conditions where the number of cases is not so low. This was studied in [36], which showed that for infectious diseases with a high transmission rate such as Covid-19, controlling the epidemic by manual contact-tracing is not feasible, because too much transmission will have occurred before exposed individuals are notified. The authors of [36] considered the use of mobile phone apps for immediate notification once a positive test result is returned, along with privacy issues surrounding such surveillence.

## Discussion

### What this paper is about

This is a theoretical paper studying the consequences of three potential responses to epidemics fueled by highly infective pathogens. We have incorporated some amount of information from Covid-19 in terms of characteristics of the virus and early responses, using the events in the spring of 2020 to motivate our study and to give it realism—without pretending that our models are a faithful representation of how the SARS-CoV-2 virus behaved or how any one country or community responded. Such models would require detailed medical knowledge on the virus and on immune responses, as well as detailed statistics on the ground within each country or city; that is far beyond the scope of the present paper. Our models are not intended to fit specific data; they are mathematical, conceptual models that we hope will shed light on how a Covid-like epidemic can affect a population, and how the responses of the population can in turn alter the course of the epidemic.

We modeled the infection and responses to the epidemic as an infinite dimensional dynamical system defined by a system of delay differential equations. Early events, from undetected transmission to the outbreak to the drastic measures that put everyday activity on pause, are described using a single set of equations. Parameters were chosen to produce results during the first several months that resembled real-world events. Our main results are about three different paths to recovery, post-lockdown and pre-vaccine. While the three strategies discussed were also inspired by real-world scenarios, our models are intended to be phenomenological only; they are examples of three very different ways to navigate the conflicting demands of returning to normal activity and keeping the virus at bay. Our aims are to offer insight into what each protocol will bring a year or two after the outbreak, based on rigorous analysis and simulations of models calibrated to perform during early stages of the epidemic.

### Summary statistics

**Fig 5:**
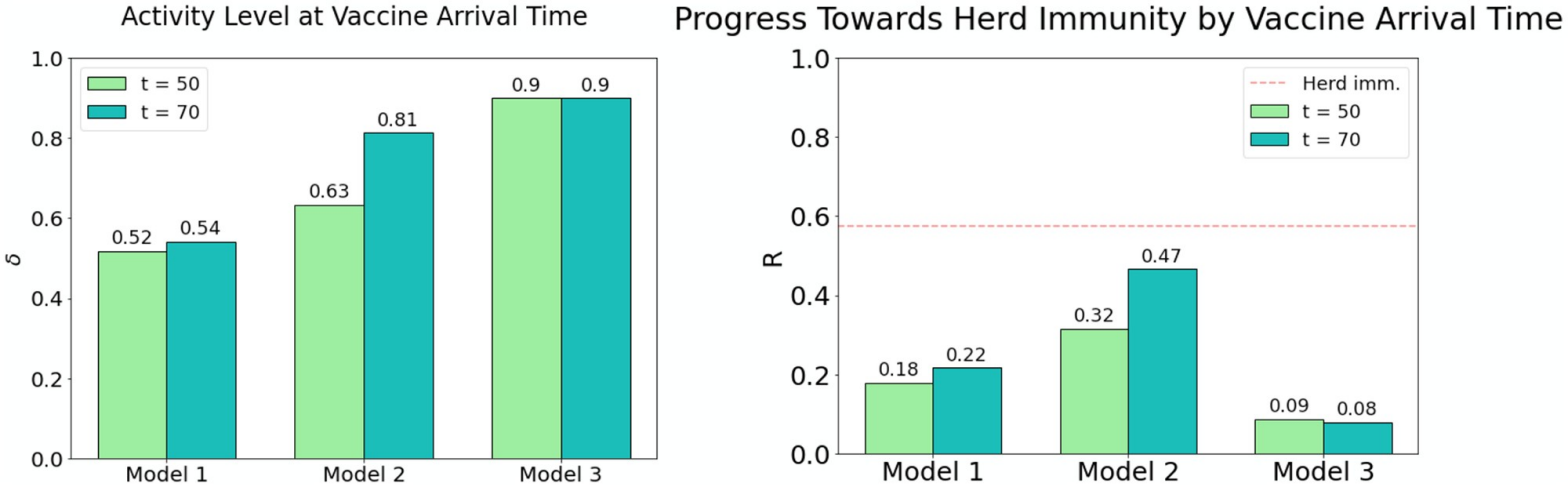
Forward projections on activity resumes and progress towards herd immunity. In the plot on the left we compare the level of activity in each of the three models at two potential vaccine arrival times (*t* = 50,70). The strategy in Model 1 allows just over 50% of normal activity by *t* = 50 and hardly makes progress by *t* = 70. Model 2 allows more than 60% of normal activity levels at *t* = 50, which increases to over 80% over the following 20 periods. Model 3 features a reopening to 90% of normal activity levels which is held constant for the duration of the simulation. The plot on the right compares the progress towards herd immunity (red dashed line) in Models 1–3. Model 1 makes steady progress towards herd immunity, but pales in comparison to Model 2, which only requires a small fraction of the population to be vaccinated to bring an end to the epidemic. In Model 3, however, the absence of new infections combined with immunity loss causes the immunity level to decline as time progresses.

Shown in Fig 5 are summary statistics comparing the three responses in terms of activity level and immunity. We evaluated the situation at two time points: at *t* = 50 and *t* = 70. For Covid-19, *t* = 50 corresponds to Spring 2021, the first time that vaccines will be widely available according to optimistic predictions. That may or may not happen, and given the myriads of challenges in vaccine distribution it will likely take a long time to reach some populations, hence we evaluate the situation again at a second time point, at *t* = 70, two years from the initial outbreak. The bar graphs in Fig 5 (left) show activity levels for the three models at *t* = 50 and 70. The ones in Fig 5(right) show *R*(*t*), the fraction of the population that is immune, at the same time points.

Statistics on *R*(*t*) have theoretical implications: They offer an estimate on the percentage of the population that must be vaccinated to reach herd immunity, i.e., for *r*_eff_ to dip below 1. For example, in order to reach 60% immunity when *R*(*t*) = 0.25 (*Q*(*t*) can be neglected as it is ≪*R*(*t*)) and the vaccine is 70% effective, it is necessary to vaccinate $35\times \frac{100}{70}=50\%$ of the population. Because the loss of immunity is assumed to be slow, *R*(*t*) can also be seen as a rough indicator of the fraction of the population that has been infected up until that point (though actual fraction is a little higher). In practice, immunity from prior infection can only be leveraged partially: While the overall magnitude of *R*(*t*) can be deduced from random serological tests and such information can be useful in policy decisions, it is harder to determine whether or not a particular individual enjoys immunity.

A glance at these bar graphs reveals that steering a conservative course designed to balance infection rate against loss of activity as described in Model 1 produces only very modest progress towards both herd immunity and economic recovery. Model 2, which takes a more aggressive stance towards reopening, accepting 3–4 times as many daily new infections, registers both higher levels of productivity and higher rates of immunity. Whether the latter is a positive or negative outcome depends on the rate of death and long-term illness among those infected. Model 3, which describes a zero-tolerance approach, beats Model 2 in the speed of economic recovery without exposing the population to the risks associated with infection. The price it has to pay is constant, unrelenting vigilance, together with the need for a more effective vaccine to reach herd immunity if this vigilance is to be relaxed.

### Insights from Models 1–3

One of the messages that emerged from our study is that even though *Q*^+^ is a poor estimate of the real level of infection, it works. A community or municipality can successfully adjust their reaction severity on an ongoing basis strictly by monitoring the number of daily new cases. However, for a virus with high asymptomatic transmission, without consistent randomized serological studies or tests that enable individuals to self-monitor with timely return of results, it would be impossible to establish a holistic understanding of the state of the epidemic.

One of the key insights from this form of modeling is understanding the timescale of the problem, which lengthens and contracts given the severity of activity reduction at any point in time. Following the growth of immunity allows a community to locate themselves within the epidemic timeline, have a clearer picture of the level of undetected infection (a call for increased testing), and more accurately respond to the resurgence of infection.

Another important point to note is that the activity reduction in our model need not be enforced top-down (requiring actual lockdowns), but can instead emerge as a result of decision making by individuals. More to that point, not every individual needs to conform to the same activity level, so long as the emergent dynamics are in line with the chosen course of infection.

From the middle of June through September 2020, the COVID-19 cases in New York City exhibited such emergent dynamics: a trajectory eerily similar to Model 1. Through a mixture of government messaging and intervention, and (most importantly) individual action, the average daily new cases in NYC remained around 300 per day for a period of almost four months [35]. Note that this was while daily new cases in the rest of the United States continued to set record highs. Starting early October, the new case count rose gradually, reaching 4,000 per day in December, a reset in *Q*^+^ similar to that in Model 2. This is likely a consequence of fatigue with COVID restrictions, holiday travel, political pressures, and failure in top-down strategy. From late fall on, the situation has been further complicated by the emergence of new variants of COVID-19 that early data show to be more easily transmittable (see Alternative Assumption (iv) below).

### Alternative assumptions

In order to convey the core ideas in a simple and clear manner, we made a number of assumptions in our models without exploring alternatives. These assumptions may or may not be valid depending on circumstance. Under different assumptions, the outcomes will likely be different, but analyses similar to those performed here may continue to apply; our methods are generalizable to wider classes of models. Below we discuss four sets of alternative assumptions:

(i) *Rate of loss of immunity.* We have assumed that once infected, immunity is robust and lasts for several years. Depending on the virus, that may or may not be true. For Covid-19, it is not known at the writing of this paper how long immunity lasts. Assuming that it lasts for at least years, a fairly safe assumption, one can continue to treat *r*_eff_(*t*) as constant locally in time in the analysis. However, shorter durations of immunity, e.g. *α* = 0.03 (i.e. immunity lasting on the order of a year) instead of 0.005 means faster return of infected individuals to the susceptible pool, and the rate of infection, which is proportional to *S*(*t*)*I*(*t*), will be higher. Also, a smaller *R*(*t*) due to immunity loss will increase the fraction that needs to be vaccinated to achieve herd immunity.
(ii) *Herd immunity and human behavior.* Recall that herd immunity is reached when *r*_eff_(*t*) dips below 1, and *r*_eff_(*t*) depends on human behavior (see Eq 8). If habits developed during the pandemic, e.g. the wearing of masks and social distancing, are relaxed—understandably so as people get fatigued by these restrictions–then *βm*(*t*), hence *r*_eff_(*t*), will rise again. Reducing the effort put into locating and isolating infected hosts will result in a lowered *p*, and that will likewise increase *r*_eff_(*t*).
  Quantatively, since *Q*(*t*) ≪ *R*(*t*), *r*_eff_ < 1 is approximately equivalent to
  $$\begin{array}{c} R\;>\;1-\frac{1}{\beta m(1-pe^{-\tau })}\;; \end{array}$$ (18)
  that is, the fraction *R* must exceed the quantity on the right side of Eq (18) for the infection to die out. If *βm*(1 − *pe*^−*τ*^) is allowed to go up, *R* will have to be larger. Now there are two ways to increase *R*: infection or vaccination. As we have seen, immunity through infection is slow even in Model 2. If herd immunity is to be achieved though vaccination, then relaxing pandemic restrictions and efforts means a larger fraction of the population needs to be vaccinated; exact relations are given in (18).
(iii) *Activity retained.* Our assumption in the model is that the amount of contact, hence the rate of transmission, scales with *δ*(*t*), the fraction of activity retained; this assumption is oversimplified. This is an area where progress has been encouraging: people have found ways to continue much of their activity in a relatively contact-free manner, by telecommuting, teaching, and conducting business remotely. That is to say, if *δ*(*t*) is defined to be the fraction of contact retained, so that as before, the tipping point is *δ* · *r*_eff_ = 1, then gradually activity lost could be modeled by ℓ(*δ*) = (1 − *δ*)^λ^, λ > 1, or some other function of *δ* smaller than 1 − *δ*. Indeed, continuing to work toward a smaller ℓ(*δ*) seems an important way to hasten economic recovery.
(iv) *Variants and mutations.* The emergence of new strains with different transmissibility properties (e.g. the so-called UK variant of Covid-19 that is fast dominating the pandemic scene in many countries in early 2021) can be modeled with an increased *β*; that is easily incorporated into our model as *β* is modeled as a function of time. If immunity to the original variant offers only partial protection against a new variant (as is suspected to the case for the variant now in Brasil), then new categories *S*′, *E*′, *I*′, *R*′ will have to be created and individuals in *R* are now in *S*′ with perhaps an attenuated *β*′. The idea of transfer between compartments with time-dependent coefficients provides very flexible dynamic modeling within homogeneous populations.

### Limitations of our models and proposed extensions

An obvious limitation is our underlying assumption of homogeneity within the population considered. (See e.g. [21, 37, 38].) We have stated that we wished to focus on large metropolitan areas (or more extended regions containing such areas), but even in this context there is a fair amount of heterogeneity in terms of demographics, such as socio-economic conditions leading to quite different amounts of activity and densities of contact. Another aspect of heterogeneity that has attracted a great deal of attention with regard to Covid is *age distribution*. Older age groups are more vulnerable as age is correlated to compromised immune systems and the presence of underlying health conditions. Social practices are also age-dependent, with the younger generation more inclined to practice the strategies detailed in Model 2.

Heterogeneity affects not only spread patterns but also responses. Partial lockdowns affecting only regions with high infection rates may be less disruptive. In agent-based models, heterogeneous responses are easily implemented via the concept of communities. In mean-field type models such as ours, a first approximation may be to divide the population into several compartments, and to simulate community structures by stipulating different degrees of contact within and across communities. This will lead to demographics-dependent reproductive numbers *r*_eff_, hence different rates of infection. We propose this as a starting point for tackling the inhomogeneous nature of transmission and response within a population.

Another very important factor not considered in this paper is *inter-regional mixing*. Unless infection rates are similar in all countries or in all regions within a country, travel of individuals from “hot spots” to calmer regions can set off spikes in infections in the latter. Requiring out-of-region travelers to quarantine for a number of days will mitigate the effect, but unless these rules are successfully enforced, new infections introduced from external sources are unavoidable and can be significant.

These extensions together with the alternative assumptions outlined above could lead to substantial generalizations treatable by methodologies very similar to those employed in this paper.
